# Evaluating dietary acidifiers as alternatives for conventional feed-based antibiotics in nursery pig diets

**DOI:** 10.1093/tas/txab040

**Published:** 2021-03-04

**Authors:** Payton L Dahmer, Cassandra K Jones

**Affiliations:** Department of Animal Sciences and Industry, Kansas State University, Manhattan, KS 66506, USA

**Keywords:** antibiotic alternative, acidifier, fecal scores, growth, nursery pig

## Abstract

A total of 360 weanling pigs (DNA 200 × 400; initially 9.7 ± 0.23 kg BW) were used in a 21-d experiment with 6 pigs/pen, 10 replicate pens/treatment, and 2 separate nursery rooms, each with 30 pens. Pigs were weighed and allotted to pens based on BW in a completely randomized block design to one of six treatment diets: 1) Negative control (no organic acids or antibiotics) and the control with 2) 0.25% acidifier A; 3) 0.3% acidifier B; 4) 0.5% acidifier C); 5) 50 g/ton carbadox; and 6) 400 g/ton chlortetracycline (**CTC**). Upon weaning, a common diet with no antibiotics or additives was fed for 21 d (Phases 1 and 2; days −21 to 0), followed by a 21-d experimental period (Phase 3; days 0 to 21) where treatment diets were fed. Pigs and feeders were individually weighed on a weekly basis to calculate average daily gain (**ADG**), average daily feed intake (**ADFI**), and feed efficiency (**G:F**). Data were analyzed using the PROC GLIMMIX procedure of SAS (v 9.4, SAS Inst., Cary, NC) with pen as the experimental unit, treatment as a fixed effect, and room as a random effect. Dietary treatment had a significant impact (*P* < 0.05) on ADG, ADFI, and G:F each week and for the overall experimental period (days 0 to 21). Specifically, from days 0 to 7, pigs fed CTC had increased (*P* = 0.001) ADG compared with those fed acidifier B, acidifier C, and carbadox, whereas pigs fed the negative control and acidifier A diets were intermediate. Additionally, pigs fed the CTC diet had improved (*P* = 0.0002) ADFI when compared with all other treatments. From days 7 to 14 and days 14 to 21, pigs fed the carbadox diet had decreased (*P* < 0.0001) ADG compared with all other treatments. During the overall period (days 0 to 21), pigs fed diets containing carbadox had reduced ADG and ADFI (*P* < 0.0001), whereas pigs fed CTC had improved (*P* < 0.0001) ADG compared with all other treatments. Additionally, blood parameters, fecal consistency, and fecal microbial populations were analyzed on a subset of pigs (*n* = 5 pigs/treatment). Dietary treatment significantly affected (*P* < 0.05) concentrations of protein, globulin, phosphorus, alkaline phosphatase, and sorbitol dehydrogenase in the blood. Treatment also significantly impacted (*P* = 0.0005) fecal score but did not affect (*P* = 0.59) fecal microbial growth from days 0 to 21. In summary, CTC continues to be a valuable additive to improve performance in the nursery. Further investigation surrounding the efficacy of dietary acidifiers as antibiotic alternatives is warranted given inconclusive evidence in this study.

## INTRODUCTION

During the transition from a liquid milk diet to solid feed, the intestinal morphology of the weanling pig drastically changes. To maximize nutrient absorption and utilization, the addition of feed additives is common in the period immediately postweaning. The stress and potential health challenges that piglets face during this time can result in negative impacts on the digestive system, which can ultimately reduce performance. Additionally, pigs tend to eat less during this time, and Le Dividich and Sève (2000) reported that an 8- to 14-d recovery period is often needed by piglets following weaning before their energy intake returns to levels, which meet their nutritional requirements. Historically, feed-based antibiotics were among the most common additives to nursery pig diets for both their therapeutic potential in disease treatment and subtherapeutic inclusion for growth promotion (Jacela et al., 2009). Antibiotics have been shown to improve growth performance by many mechanisms, including suppressing the growth of pathogenic bacteria and increasing the digestion and utilization of nutrients through the intestinal wall (Gaskins et al., 2002). In addition to antibiotics, the use of pharmacological levels of Zn and Cu can effectively treat and control postweaning diarrhea and improve growth performance in the nursery (Shelton et al., 2011; Coble et al., 2017). Despite these benefits, there is consumer and regulatory pressure to limit their use given concerns over the development of antimicrobial-resistant bacteria in humans or negative environmental impacts (Bager et al., 2000; Jondreville et al., 2003). Thus, animal scientists are actively investigating biological alternatives for these conventional antimicrobials.

Many other feed additives, such as probiotics, oligosaccharides, sea plants, spices, and herbs, have been studied as potential antibiotic alternatives, but their efficacy is variable (Turner et al., 2001). Data suggest that there is potential for dietary acidifiers to provide prophylactic effects similar to antibiotics, specifically by limiting the growth of harmful enteric pathogens and simultaneously allowing beneficial organisms to proliferate (Kim et al., 2005). Acidifiers are compounds typically classified as organic or inorganic acids and can improve growth performance by reducing or stabilizing gastric pH, ultimately increasing nutrient digestibility, and limiting the growth of pathogenic bacteria (Jacela et al., 2009). It has also been established that this reduction in gastric pH helps to increase gastric proteolysis and nutrient absorption through the intestinal wall, while subsequently limiting the growth of negative bacteria in the gut (Roth and Kirchgessner, 1998). This ultimately allows them to counteract some of the detrimental effects of the postweaning period. Although the increase in buffering capacity of the pig’s gut has been reported as a primary mechanism of organic acids, many studies suggest that their mode of action extends well beyond this. Roth and Kirchgessner (1998) describe that various organic acids can also improve protein and energy digestibility, alter gastrointestinal bacterial populations, and work as antimicrobial agents, suggesting that their mode of action is multifunctional.

Although acidifiers have been heavily evaluated in recent years, very few studies directly compare these products under controlled conditions. Most dietary acidifiers are used as blends of acids, and the response of these products depends on the inclusion level, components of types of acids included, and other nutritional the diet. Based on previous studies, organic acids have proven more beneficial to growth performance of weanling pigs when compared with inorganic acids (Kil et al., 2011; Liu et al., 2018). Although literature is generally supportive of organic acids improving nursery pig growth performance, little direct comparison of commercial acidifier blends and commonly used antimicrobials is available with economic application, limiting producers’ ability to make relevant, science-based decisions to include them. Thus, the objective of this experiment was to evaluate three commercially available dietary acidifiers and their impacts on nursery pig growth performance, fecal score, fecal microbial populations, and blood serum metabolites when compared with two commonly used feed-based antibiotics.

## MATERIALS AND METHODS

All experimental procedures adhered to guidelines for the ethical and humane use of animals for research according to the Guide for the Care and Use of Agricultural Animals in Research and Teaching (FASS, 2010) and were approved by the Institutional Animal Care and Use Committee at Kansas State University (IACUC #4036.31).

### Animals and Diets

A total of 360 weanling pigs (DNA 200 × 400; initially 9.4 ± 0.23 kg BW; approximately 21 d old) were utilized in a 21-d experiment at the Kansas State University Swine Teaching and Research Facility (Manhattan, KS). Upon weaning, pigs were individually weighed, tagged, and allotted to pens according to BW in a completely randomized block design. Blocking was completed by utilizing two separate environmentally controlled nursery rooms, each with 30 pens. Each pen (1.52 × 1.52 m) included a four-hole dry self-feeder and a nipple waterer to provide pigs ad libitum access to feed and water. A total of six pigs were placed into each of the 60 pens (10 replicate pens per treatment) and randomly assigned to one of six dietary treatments: 1) Negative control (no organic acids or antibiotics) and the control with 2) 0.25% acidifier A (KEM-GEST, Kemin Industries, Des Moines, IA); 3) 0.3% acidifier B (ACTIVATE DA, Novus International, Saint Charles, MO); 4) 0.50% acidifier C (OutPace, PMI Additives, Arden Hills, MN); 5) 50 g/ton carbadox (Mecadox 10, Phibro Animal Health, Teaneck, NJ, or 6) control + 400 g/ton chlortetracycline (**CTC**; Deracin 100, PharmGate Animal Health, Wilmington, NC). The acidifiers used represent a variety of commonly used acid blends, and all inclusion levels were based on the manufacturer’s recommendations. The CTC was included at therapeutic levels based on a veterinary feed directive, whereas carbadox was included at subtherapeutic levels in the diet. Upon weaning, pigs were fed common Phase 1 and Phase 2 starter diets without antimicrobials or acidifiers for 21 d and then fed experimental Phase 3 diets for 21 d. The transition to Phase 3 diets was considered day 0 of the experiment. All diets were formulated to meet or exceed NRC (2012) nutrient requirements. Treatments consisted of a standard corn- and soybean meal-based diet, whereas addition of dietary acidifiers or medications was included by the substitution of corn. Diets were manufactured by Hubbard Feeds (Hubbard Feeds, Beloit, KS) and were fed in pellet form during the common feed period and meal form during the experimental period (Table 1).

**Table 1.** Diet composition (as-fed basis)^1^

		Dietary treatment^2^				
	Control	Acidifier A	Acidifier B	Acidifier C	Carbadox	Chlortetracycline
Ingredient, %						
Corn	65.69	65.34	65.44	65.09	64.9	65.39
Soybean meal, 46.5% CP	30.20	30.20	30.20	30.20	30.20	30.20
Calcium carbonate	1.00	1.00	1.00	1.00	0.66	1.00
Limestone phosphate, 21%	0.95	0.95	0.95	0.95	0.95	0.95
Sodium chloride	0.58	0.58	0.58	0.58	0.58	0.58
l-Lysine	0.55	0.55	0.55	0.55	0.55	0.55
dl-Methionine	0.27	0.27	0.27	0.27	0.27	0.27
l-Threonine	0.25	0.25	0.25	0.25	0.25	0.25
l-Tryptophan	0.07	0.07	0.07	0.07	0.07	0.07
l-Valine	0.14	0.14	0.14	0.14	0.14	0.14
Trace mineral premix^3^	0.15	0.15	0.15	0.15	0.15	0.15
Vitamin w/ phytase^4^	0.25	0.25	0.25	0.25	0.25	0.25
Experimental ingredient	N/A	0.25	0.3	0.5	1.00	0.2
Total	100.0	100.0	100.0	100.0	100.0	100.0
Calculated analysis						
Standardized ileal digestibility (SID) amino acids, %						
Lys	1.33	1.33	1.33	1.33	1.33	1.33
Ile:Lys	51	51	51	51	51	51
Leu:Lys	107	107	107	107	107	107
Met:Lys	38	38	38	38	38	38
Met and Cys:Lys	58	58	58	58	58	58
Thr:Lys	63	63	63	63	63	63
Trp:Lys	20	20	20	20	20	20
Val:Lys	69	69	69	69	69	69
Total Lys, %	1.47	1.47	1.47	1.47	1.47	1.47
Metabolizable energy, kcal/kg	3,264	3,255	3,258	3,247	3,242	3,258
Net energy, kcal/kg	2,320	2,313	2,315	2,306	2,302	2,316
CP, %	20.2	20.2	20.2	20.2	20.2	20.2
Ca, %	0.74	0.74	0.74	0.74	0.75	0.74
P, %	0.58	0.59	0.58	0.57	0.57	0.58
Available P, %	0.29	0.31	0.29	0.29	0.29	0.29

^1^A total of 360 weanling pigs (DNA 200 × 400) were used in a three-phase nursery trial with 6 pigs per pen and 10 replicates per treatment. A common diet was fed from day −21 to day 0 (Phases 1 and 2). Treatment diets were fed from days 0 to 21 (Phase 3).

^2^Diets included either 0.25% acidifier A (KEM-GEST, Kemin Industries, Des Moines, IA); 0.3% acidifier B (ACTIVATE DA, Novus International, Saint Charles, MO); 0.5% acidifier C (OutPace, PMI Additives, Arden Hills, MN); 50 g/ton carbadox (Mecadox 10, Phibro Animal Health, Teaneck, NJ); or 400 g/ton chlortetracycline (Deracin 100, Pharmgate Animal Health, Wilmington, NC).

^3^Provided per kilogram of premix: 22 g Mn from manganese oxide; 73 g Fe from iron sulfate; 73 g Zn from zinc sulfate; 11 g Cu from copper sulfate; 198 mg I from calcium iodate; and 198 mg Se from sodium selenite.

^4^Provided per kilogram of premix: 750,000 IU vitamin A; 300,000 IU vitamin D3; 8,000 IU vitamin E; 1,500 mg riboflavin; 5,000 mg pantothenic acid; 9,000 mg niacin; and 6 mg vitamin B12.

### Chemical Analysis of Diets

Complete diet samples were collected from 10 different feeders per dietary treatment on days 0 and 21, and composite subsamples were submitted for chemical analysis (Midwest Laboratories, Omaha, NE). Assays included dry matter (method 930.15; AOAC, 2007), crude protein (**CP**) as N × 6.25 using the combustion method (Nitrogen Determinator; model TruMac N, Leco Corporation, St. Joseph, MI; method 990.03; AOAC, 2007), acid detergent fiber (**ADF**; ANKOM Tech. Method 200), Ca (AOAC 985.01, 2006), P (AOAC 985.01, 2006), and metabolizable energy (**ME**) by calculation (Table 2).

**Table 2.** Chemical analysis of experimental diets^1^

	Dietary treatment^2^					
Item	Control	Acidifier A	Acidifier B	Acidifier C	Carbadox	Chlortetracycline
Day 0						
Dry matter, %	86.0	86.2	86.7	86.7	86.6	86.6
Crude protein, %	20.4	20.7	20.0	20.2	19.6	21.6
Acid detergent fiber, %	3.6	3.8	3.6	3.3	4.0	3.3
Ca, %	0.74	0.78	0.84	0.7	0.59	0.74
P, %	0.67	0.63	0.65	0.57	0.55	0.60
Metabolizable energy, kcal/kg	1,310	1,300	1,310	1,320	1,320	1,300
Day 21						
Dry matter, %	87.3	87.0	87.6	87.3	87.2	87.3
Crude protein, %	20.1	22.8	16.5	21.0	21.0	20.2
Acid detergent fiber, %	3.5	6.2	4.8	5.0	4.2	3.6
Ca, %	0.83	1.00	1.05	0.97	0.76	0.75
P, %	0.63	0.85	0.61	0.81	0.60	0.64
Metabolizable energy, kcal/kg	1,340	1,270	1,350	1,300	1,320	1,340

^1^Complete diet samples were obtained from each dietary treatment on day 0 and day 21, representing at least 10 different samples per diet. Samples of diets were pooled and analyzed for dry matter, crude protein, acid detergent fiber, Ca, P, and metabolizable energy (Midwest Laboratories Inc., Omaha, NE).

^2^Diets included either 0.25% Acidifier A (KEM-GEST, Kemin Industries, Des Moines, IA); 0.3% Acidifier B (ACTIVATE DA, Novus International, Saint Charles, MO); 0.5% Acidifier C (OutPace, PMI Additives, Arden Hills, MN); 50 g/ton carbadox (Mecadox 10, Phibro Animal Health, Teaneck, NJ); or 400 g/ton chlortetracycline (Deracin 100, Pharmgate Animal Health, Wilmington, NC).

### Data Collection (Growth Performance, Blood Sampling, and Fecal Swabbing/Scoring)

All pigs were weighed individually on days 0 and 21, and pen weights were collected utilizing a floor scale on days 7 and 14. Feeders from each pen were individually weighed on days 0, 7, 14, and 21 to record feed disappearance. Average daily gain (**ADG**) and average daily feed intake (**ADFI**) were calculated on a weekly basis. Whole blood samples were collected from the same 30 pigs (five pigs per treatment) on days 0 and 21 of the experiment. Blood was collected from the jugular vein by venipuncture using a sterile 3-mL vacuum-sealed tube. Following collection, samples were placed on ice and immediately transported to the Kansas State University Veterinary Diagnostic Laboratory (Kansas State University, Manhattan, KS) for a complete blood panel, serum chemistry, and hepatic profile via spectrophotometry. Briefly, samples were centrifuged for 5 min at 3,000 rpm (Eppendorf North America, Enfield, CT) to separate the serum for analysis. Chemistry assays were then performed utilizing the Cobas c501 (Roche Diagnostics, Indianapolis, IN).

Additionally, fecal samples were collected from 30 pigs (five pigs per treatment) on days 0 and 21 for analysis of enteric bacteria and antimicrobial resistance. Samples were analyzed by the Iowa State University Veterinary Diagnostic Laboratory (Iowa State University, Ames, IA) for bacterial isolation and identification. Samples were collected aseptically utilizing sterile cotton-tipped collection swabs (Copan Diagnostics, Murrieta, CA) by rectal massage and stored in transport tubes with reduced oxygen at 4 °C until analyzed. Samples were then plated without incubation or enrichment on selective media and incubated at 37 °C for 24 h as described by the FDA Bacteriological Analytical Manual (Drug Administration, 1998). Suspect colonies were serogrouped for final identification. Bacterial colonies were then tested for antimicrobial susceptibility by comparing a modified minimum inhibitory concentration to a susceptibility breakpoint as described by Brooks et al. (2003). Fecal scoring was also conducted by two independent, trained scorers on days 0, 1, 2, 7, 14, and 21 to categorize the consistency of piglet feces per pen. A numerical scale from 1 to 5 was used: 1) being hard pellet-like feces, 2) a firm formed stool, 3) a soft moist stool that retains shape, 4) a soft unformed, and 5) a watery liquid stool (Tables 3–5).

**Table 3.** Effects of dietary treatment on nursery pig growth performance^1^

Item	Control	Acidifier A	Acidifier B	Acidifier C	carbadox	Chlortetracycline	SEM	Treatment	Medicated vs. none	Acidifier vs. none
BW, kg										
Day 0	9.4	9.7	9.6	9.9	9.5	10.1	0.23	0.129	0.074	0.102
Day 7	12.3^b^	12.8^ab^	12.5^b^	12.8^ab^	12.3^b^	13.7^a^	0.25	0.001	0.026	0.165
Day 14	16.2^bc^	17.0^b^	16.7^bc^	17.1^ab^	15.4^c^	18.4^a^	0.33	<0.0001	0.102	0.069
Day 21	22.0^b^	22.4^b^	22.4^b^	22.7^b^	19.5^c^	24.6^a^	0.39	<0.0001	0.904	0.233
(Days 0 to 7)										
ADG, kg/d	0.45^ab^	0.47^ab^	0.43^b^	0.41^b^	0.39^b^	0.52^a^	0.035	0.001	0.766	0.649
ADFI, kg/d	0.61^b^	0.62^b^	0.60^b^	0.63^b^	0.62^b^	0.70^a^	0.022	0.0002	0.007	0.839
G:F	0.74^ab^	0.75^a^	0.71^ab^	0.66^ab^	0.63^b^	0.75^a^	0.038	0.007	0.189	0.389
(Days 7 to 14)										
ADG, kg/d	0.55^b^	0.58^ab^	0.60^ab^	0.62^ab^	0.45^c^	0.67^a^	0.021	<0.0001	0.701	0.037
ADFI, kg/d	0.81^ab^	0.84^ab^	0.80^b^	0.89^ab^	0.75^b^	0.95^a^	0.039	0.002	0.415	0.459
G:F	0.68^ab^	0.70^ab^	0.80^a^	0.70^ab^	0.61^b^	0.70^ab^	0.062	0.050	0.604	0.255
(Days 14 to 21)										
ADG, kg/d	0.80^b^	0.78^b^	0.81^ab^	0.80^b^	0.58^c^	0.89^a^	0.020	<0.0001	0.007	0.816
ADFI, kg/d	1.09^b^	1.07^bc^	1.09^b^	1.16^ab^	0.94^c^	1.26^a^	0.036	<0.0001	0.911	0.758
G:F	0.74^a^	0.74^a^	0.74^a^	0.69^ab^	0.62^b^	0.71^b^	0.023	0.001	0.009	0.614
(Days 0 to 21)										
ADG, kg/d	0.60^b^	0.61^b^	0.61^b^	0.61^b^	0.47^c^	0.69^a^	0.015	<0.0001	0.349	0.548
ADFI, kg/d	0.84^bc^	0.84^bc^	0.83^bc^	0.89^ab^	0.77^c^	0.97^a^	0.033	<0.0001	0.282	0.537
G:F	0.72^a^	0.73^a^	0.74^a^	0.69^a^	0.62^b^	0.72^a^	0.017	<0.0001	0.004	0.994
Feed cost, $/kg feed^3^	0.06	0.06	0.06	0.06	0.07	0.06	—	—	—	—
Feed cost, $/pig^4^	1.05^c^	1.08^bc^	1.07^bc^	1.17^ab^	1.08^bc^	1.27^a^	0.044	<0.0001	0.001	0.085
Feed cost, $/kg gain^5^	1.05^c^	1.08^bc^	1.07^bc^	1.17^ab^	1.08^bc^	1.27^a^	0.044	<0.0001	0.001	0.085
Income over feed^6^	3.02^b^	2.99^b^	3.15^b^	3.14^b^	2.43^c^	3.51^a^	0.099	<0.0001	0.580	0.352

^1^A total of 360 weanling pigs (6 pigs per pen, 10 pens per treatment) were fed a common diet during Phase 1 and Phase 2 with treatment diets fed during Phase 3.

^2^Diets included either 0.25% acidifier A (KEM-GEST, Kemin Industries, Des Moines, IA); 0.3% acidifier B (ACTIVATE DA, Novus International, Saint Charles, MO); 0.5% acidifier C (OutPace, PMI Additives, Arden Hills, MN); 50 g/ton carbadox (Mecadox 10, Phibro Animal Health, Teaneck, NJ); or 400 g/ton chlortetracycline (Deracin 100, Pharmgate Animal Health, Wilmington, NC).

^3^Calculated using ingredient prices as of April 28, 2020.

^4^Feed cost, $/pig = feed cost per kg of feed × (ADFI overall × 21).

^5^Feed cost, $/kg of gain = feed cost per pig ÷ (ADG overall × 21).

^6^Income over feed = [$0.25 × (day 21 BW − day 0 BW)] − feed cost per pig.

^abc^Means within a row that do not share a common superscript differ *P* > 0.05.

**Table 4.** Impact of dietary treatment on nursery pig blood parameters on day 21^1^

	Dietary treatment^2^							
Item	Control	Acidifier A	Acidifier B	Acidifier C	Carbadox	Chlortetracycline	SEM	*P*-value
Glucose, mg/dL	118.40	112.50	111.90	112.10	100.80	114.60	4.614	0.14
Urea nitrogen^x^	7.30	6.80	7.70	7.67	9.80	9.60	0.886	0.08
Creatinine, mg/dL^x^	0.68	0.76	0.82	0.75	0.83	0.82	0.056	0.19
Protein, g/dL^x^	5.00^ab^	5.00^ab^	5.30^ab^	5.00^ab^	5.40^a^	4.90^b^	0.146	0.01
Albumin, g/dL^x^	3.59	3.66	3.53	3.38	3.50	3.67	0.125	0.33
Globulin, g/dL^x^	1.41^bc^	1.30^c^	1.73^ab^	1.62^abc^	1.94^a^	1.26^c^	0.108	<0.0001
Calcium, mg/dL^xy^	11.06	11.04	11.06	11.04	10.93	10.86	0.172	0.88
Phosphorus, mg/dL^x^	10.26^a^	10.61^a^	10.04^a^	10.48^a^	7.98^b^	10.61^a^	0.397	<0.0001
Sodium, mmol/L^x^	142.7	144.00	142.42	142.80	142.12	143.40	0.896	0.68
Potassium, mmol/L^x^	6.93	6.78	7.09	6.60	6.24	7.00	0.363	0.47
Chloride, mmol/L	90.10	100.75	99.25	100.50	99.50	100.60	4.483	0.40
Bicarbonate, mmol/L^x^	24.36	22.75	22.00	24.78	24.40	24.66	1.574	0.43
Anion gap, mmol/L^x^	26.30	28.38	29.50	25.31	25.76	26.30	1.864	0.18
Na:K^x^	21.60	21.50	20.50	21.90	23.38	20.80	0.940	0.27
Aspartate transaminase P5P, U/L	70.80	74.13	106.83	58.40	128.00	89.30	27.379	0.42
Alkaline phosphatase, U/L^y^	390.90^ab^	299.25^b^	359.17^ab^	421.99^ab^	498.74^a^	394.70^ab^	56.204	0.04
Gamma glutamyltransferase^x^	60.55	45.88	54.50	61.46	67.01	68.65	9.701	0.26
Sorbitol dehydrogenase, U/L^xy^	1.41^b^	0.76^b^	0.52^b^	0.44^b^	24.48^a^	0.31^b^	5.552	0.02

^1^A total of 30 whole blood samples (5 pigs/treatment) were collected on day 0 and day 21 of the experiment and submitted to the Kansas State University Veterinary Diagnostic Laboratory (Kansas State University, Manhattan, KS).

^2^Diets included either 0.25% acidifier A (KEM-GEST, Kemin Industries, Des Moines, IA); 0.3% acidifier B (ACTIVATE DA, Novus International, Saint Charles, MO); 0.5% Acidifier C (OutPace, PMI Additives, Arden Hills, MN); 50 g/ton carbadox (Mecadox 10, Phibro Animal Health, Teaneck, NJ); or 400 g/ton chlortetracycline (Deracin 100, Pharmgate Animal Health, Wilmington, NC).

^abc^Means within a row that do not share a common superscript differ P > 0.05. Values reported are least square means, representing the main effects of dietary treatment.

^x^Main effect of day is significant (*P* < 0.05).

^y^Interaction of treatment × day is significant (*P* < 0.05).

**Table 5.** Impact of dietary treatment on nursery pig average fecal score and fecal microbial growth

	Dietary treatment^1^							*P*-value		
Item	Control	Acidifier A	Acidifier B	Acidifier C	carbadox	Chlortetracycline	SEM	Treatment	Day	Treatment × Day
Average Fecal Score^2^	3.2^a^	3.2^a^	3.2^a^	3.2^a^	2.9^b^	3.2^a^	0.05072	0.0005	<0.0001	0.11
Average Microbial Growth^3^	3.37	3.60	3.47	3.44	3.23	3.38	0.144	0.59	0.002	0.47

^1^Diets included either 0.25% acidifier A (KEM-GEST, Kemin Industries, Des Moines, IA); 0.3% acidifier B (ACTIVATE DA, Novus International, Saint Charles, MO); 0.5% acidifier C (OutPace, PMI Additives, Arden Hills, MN); 50 g/ton carbadox (Mecadox 10, Phibro Animal Health, Teaneck, NJ); or 400 g/ton chlortetracycline (Deracin 100, Pharmgate Animal Health, Wilmington, NC).

^2^Fecal scores were collected on days 0, 1, 2, 7, 14, and 21 of the experiment by two trained, independent scorers using a numerical scale: 1 = hard, pellet-like feces; 2 = firm, formed stool; 3 = soft, moist stool that retains shape; 4 = soft, unformed stool; 5 = watery, liquid stool.

^3^Fecal samples from 30 pigs (5 pigs per treatment) were collected on days0 and 21 via rectal swab and plated for analysis of enteric bacteria by the Iowa State University Veterinary Diagnostic Laboratory (Iowa State University, Ames, IA). Culture growth from day 0 to day 21 was reported using a numeric scale: 0 = no significant growth; 1 = low; 2 = few; 3 = moderate; 4 = high.

^abc^Means within the same row that do not share a common superscript differ *P* < 0.05. Values reported are least square means, representing the main effect of dietary treatment.

### Statistical Analysis

Data were analyzed as a completely randomized block design using the PROC GLIMMIX procedure of SAS Studio (version 9.4, SAS Institute, Inc., Cary, NC), with pen as the experimental unit. Treatment was included as a fixed effect, and room was included as a random effect in the statistical model. All comparisons incorporated Tukey−Kramer multiple comparison adjustments. Preplanned pairwise contrasts were also utilized to compare medicated diets and none (CTC or carbadox vs. control) as well as organic acid diets and none (acidifier A, acidifier B, or acidifier C vs. control). Results were considered significant if *P* ≤ 0.05 and a trend if 0.05 > *P* ≤ 0.10.

## RESULTS

### Nursery Pig Growth Performance

Dietary treatment had a significant effect (*P* < 0.05) on nursery pig ADG, ADFI, and G:F in each phase and for the overall experiment (days 0 to 21). From days 0 to 7, pigs fed the diet containing CTC had improved (*P* = 0.001) ADG compared with those fed diets with acidifier B, acidifier C, or carbadox, whereas pigs fed the control or acidifier A treatments were intermediate. Additionally, ADFI was greater (*P* = 0.0002) for pigs fed the CTC diet when compared with those fed all other treatments. Feed efficiency was improved (*P* = 0.007) for those pigs fed the CTC or acidifier A diets when compared with pigs fed carbadox and pigs fed the control and diets containing acidifiers B or C were intermediate.

From days 7 to 14, pigs fed the CTC diet had improved (*P* < 0.0001) ADG compared with those fed the control or carbadox diets. Pigs consuming the three acidifier blend diets were intermediate. Feed intake was increased (*P* = 0.002) for pigs fed the CTC diet when compared with pigs fed acidifier B or carbadox, with the remaining treatments being intermediate. Differences in G:F across treatments during this period were significant (*P* = 0.05) where pigs fed acidifier B had improved feed efficiency compared with those fed carbadox, and the remaining treatments were intermediate.

During the final week of the experiment (days 14 to 21), ADG was greatest (*P* < 0.0001) for pigs fed the CTC diet and poorest for pigs fed the carbadox diet. Again, ADFI was the highest (*P* < 0.0001) for pigs fed the CTC treatment and lowest for those fed the carbadox diet. Feed efficiency was greater (*P* = 0.001) for pigs fed the control, acidifier A, and acidifier B diets when compared with those fed carbadox, with pigs fed the acidifier C treatment being intermediate.

Finally, during the overall experiment (days 0 to 21), ADG was the greatest (*P* < 0.0001) for pigs fed CTC when compared with all other treatments. Likewise, ADFI was increased (*P* < 0.0001) for pigs fed the CTC diet when compared with those fed the control, acidifier A, acidifier B, and carbadox diets, whereas those fed acidifier C were intermediate. Feed efficiency was decreased (*P* < 0.0001) for pigs fed the carbadox treatment when compared with those on all other diets. There was no evidence for differences (*P* = 0.129) in piglet BW on day 0 of the experiment; however by day 7, pigs fed CTC were heavier (*P* = 0.001) compared with those fed the control, acidifier B, or carbadox treatments. Thus, by the end of the 21-d experiment, pigs fed CTC were the heaviest (*P* < 0.0001) and those fed carbadox were the lightest.

### Economic Application

Feed costs were calculated for dollars per kilogram of feed, dollars per pig, and dollars per kilogram of gain utilizing current ingredient prices. Income over feed (IOF) was also calculated by subtracting the feed cost per pig from a predicted revenue. The predicted revenue was a fixed amount, set at $0.25 per kg of gain, taking into consideration the current market price at the time of the experiment. This calculation was done on a per pen basis to have proper replication for statistical analysis. Economic data were included in the statistical model previously described.

Given all treatment diets were formulated from the control, differences in the cost of each diet depend solely on the price of the additive included. When compared with the control diet, which was the least expensive, the diet including carbadox was the most expensive per kilogram of feed (Feed Cost, $/kg of feed: $0.0596 and $0.0666, respectively). Feed cost per pig was calculated as follows: Feed Cost, $ per pig = Feed Cost, $ per kg of feed × (ADFI Overall × 21). Feed cost per kg of gain was also calculated as follows: Feed cost, $ per kg of gain = Feed Cost, $ per pig ÷ (ADG Overall × 21) . Significant differences in feed cost, both per pig and per kilogram of gain, were observed across treatments (*P* < 0.0001). Although costs associated with feeding the diet containing CTC were statistically higher (*P* < 0.0001), IOF calculations determined that the margin of profit is potentially greater (*P* < 0.0001) by including CTC in the diet when compared with the other additives used in this study.

### Nursery Pig Blood Parameters, Fecal Consistency, and Fecal Microbial Population

From days 0 to d 21, dietary treatment significantly impacted (*P* < 0.05) the concentrations of protein, globulin, phosphorus, alkaline phosphatase, and sorbitol dehydrogenase in nursery pig blood. A main effect of day was also observed, whereas mean values for urea nitrogen and globulin were lower (*P* < 0.05) at day 21 compared with day 0, and mean values for creatinine, protein, albumin, phosphorus, bicarbonate, anion gap, calcium, sodium-potassium ratio, and sorbitol dehydrogenase were significantly higher (*P* < 0.05) at day 21 compared with day 0. The only blood parameters for which a significant treatment × day interaction was observed (*P* ≤ 0.03) were calcium, alkaline phosphatase, and sorbitol dehydrogenase.

Blood data indicate that pigs fed CTC had lower total protein concentrations (*P* = 0.01) compared with those fed carbadox, whereas the remaining treatments were intermediate. Globulin levels were increased (*P* < 0.0001) in pigs fed the carbadox treatment compared with those fed CTC, acidifier A, or the negative control. The pigs consuming carbadox also showed increased (*P* = 0.04) alkaline phosphatase concentrations compared to pigs fed acidifier A, whereas other dietary treatments were intermediate. Finally, pigs fed carbadox had significantly increased (*P* = 0.02) levels of sorbitol dehydrogenase when compared with pigs consuming all other treatments.

### Nursery Pig Fecal Consistency and Gut Microflora

For the duration of the experiment, there was no evidence (*P* = 0.11) of a significant dietary treatment × day interaction with regard to fecal score. However, the main effect of treatment significantly impacted fecal score (*P* = 0.0005), with a mean fecal score of 3.2 for pigs fed the negative control, acidifier A, acidifier B, acidifier C, and CTC. This indicates that pigs fed the carbadox treatment had a lower average fecal score throughout the experiment when compared with all other diets, suggesting that these pigs had firmer feces when compared with their contemporaries. Additionally, fecal score was also impacted by sampling day (*P* < 0.0001), with mean scores of 3.1, 3.1, 3.0, 3.2, 3.3, and 3.3 for days 0, 1, 2, 7, 14, and 21, respectively.

No impact (*P* = 0.59) was observed by dietary treatment on nursery pig fecal microbial growth, with mean growth values of 3.37, 3.60, 3.47, 3.44, 3.23, and 3.38 reported for the negative control, acidifier A, B, C, carbadox, and CTC, respectively. However, the main effect of day (*P* = 0.0016) indicated that the growth of enteric bacteria was reduced from day 0 to day 21 (day 0 average growth = 3.6; day 21 average growth = 3.2).

## DISCUSSION

Research has demonstrated that ADG, ADFI, and G:F of weanling pigs can be enhanced by the addition of feed-based antibiotics (Zimmermann, 1986; Cromwell, 2002). Similar to previous research, the overall ADG of pigs in the current study fed diets containing CTC was greatest when compared with those fed a control or diets with commercial acidifiers. Interestingly, the addition of carbadox to the diet negatively affected ADG and G:F. Although this response was not expected, others have reported this in literature (Walsh et al., 2007).

It is known that during the period immediately postweaning, the immature digestive systems of pigs are not yet adapted to diet changes and environment, therefore apparent decreases in HCl secretion within the stomach allows rapid proliferation of harmful bacteria (Kidder and Manners, 1978). Thus, pigs experience reduced feed intake, suppressed weight gain, and diarrhea, which pose potential economic loss for swine producers. As a result, organic acids are typically most beneficial when fed within a few weeks of weaning (Roth and Kirchgessner, 1998). The current work fed a common diet to all pigs for 21 d immediately following weaning (Phase 1 and Phase 2 of the nursery) and followed this with a 21-d experimental period (Phase 3 of the nursery). Although the common diet allowed pigs a longer acclimation period postwean, waiting until Phase 3 to introduce the acidifiers could explain the lack of response observed in the trial. Future work should introduce dietary acidifiers earlier in the nursery to evaluate their efficacy.

In previous studies, fumaric and citric acids have typically been the most widely investigated. Meanwhile, the current work evaluated a larger variety of acids, specifically blends of acids in the form of commercial feed additives. One constant among previous studies and the current is the variability in nursery pig response to different acids. Ravindran and Kornegay (1993) described that causes for this variability could be linked to the type and dose of acids included, other nutritional components of the diet, or the age and existing performance of the pigs.

A review by Partanen and Mroz (1999) compiled data from 35 experiments and summarized a slight improvement in both ADG and G:F in weaned pigs supplemented with increasing levels of formic, fumaric, and citric acids compared with a control diet without acidification. However, the data did not provide evidence of an optimal inclusion level or significant differences in performance between these acids. Likewise, the current experiment did not observe differences between the organic acid treatments. Despite this, similar ADG and G:F was observed by the second week of the experiment in pigs supplemented with organic acids when compared with those fed the antibiotic CTC. Unfortunately, these differences were no longer apparent by the end of the trial. This coincides with previous research, which suggests that the ability of organic acids to promote growth performance is limited when compared with antibiotics (Petersen and Oslage, 1982). Interestingly, some studies indicate that organic acids can actually improve the absorption of antibiotics and boost their therapeutic effects when the two additives are used together (Edmonds et al., 1985), but the current study provided the two in separate treatments, so this interaction was not observed. However, data from this study do show that of the acidifiers evaluated, acidifier C was the only product that yielded similar ADFI to the leading treatment, CTC. McManus et al. (2017) fed the same product, acidifier C, at a rate of 1.3 kg/ton with the inclusion of CTC and found improved growth performance when the combination was fed.

Another factor that could explain the lack of performance differences between acidifiers in our experiment are the changes in feed intake. Previously, improvements in the growth of weaned pigs fed diets with acidifiers has been credited to enhanced palatability (Cole et al., 1968; Bouldan et al., 1988), and literature strongly indicates that feed intake in weanling pigs is extremely variable among different organic acids. The review by Partanen and Mroz (1999) describes that typically, formic acid has a positive effect, fumaric acid has no effect, and citric acid has a negative effect on feed intake. Therefore, it is necessary to consider the ingredients in commercial organic acid products and how they can affect palatability. For example, acidifier A primarily contains a blend of phosphoric, fumaric, citric, and lactic acids. A study by Henry et al. (1985) allowed free choice of two diets to weanling pigs: a control with no acidifier and a diet acidified with both citric and fumaric acids. A significant increase in feed intake was observed for the control diet, suggesting a negative palatability affect associated with citric and fumaric acids. In our study, no difference in ADFI was observed between pigs fed a diet containing acidifier A and a control, or those fed acidifier A and carbadox. Interestingly, pigs fed a diet containing acidifier A had improved ADG when compared with those fed carbadox, suggesting potential merit in this acidifier blend as an antibiotic alternative. This agrees with findings from Walsh et al. (2007), where pigs fed a diet with 0.2% acidifier A had similar growth performance to pigs fed carbadox.

Additionally, acidifier B is a combination of organic acids and 2-hydroxy-4-methylthio butanoic acid (**HMTBa**). The compound HMTBa is a methionine (**MET**) hydroxy analog, structured very similar to MET itself, and is proposed to have antimicrobial properties. In this experiment, no improvements in nursery pig ADG or ADFI were observed in pigs fed acidifier B when compared to pigs fed CTC. However, G:F was similar between pigs across these two treatments, suggesting that nutrient absorption could be similar among the two products. A study by Jendza et al. (2011) reported increased ileal digestibility of both CP and fiber in pigs fed a diet with HMBTa as the source of MET, as a result of more rapid absorption of HMBTa by the pig. A notable difference between these two experiments is the composition of diets. Jendza fed higher fiber diets with wheat middlings, whereas the current study fed standard corn-soybean meal -based diets. This suggests that future work should evaluate products like acidifier B, which contain HMBTa, and their ability to impact nutrient digestion and utilization relative to antibiotics with more uniform diet composition.

Although our experiment did not see significant differences in fecal microbial populations among dietary treatments, previous research has indicated that dietary acidifiers can positively impact the pig’s gut microbiota. The mode of action by which this is achieved is not precise, but literature suggests that undissociated forms of organic acids can diffuse across the cell membrane of pathogens, damage their cytoplasm, and hinder growth (Mroz, 2005). Research by Long et al. (2018) fed pigs two blends of organic acids, collectively containing formic, acetic, propionic, butyric, and sorbic acids and found that these acids reduced fecal *Escherichia coli* counts and subsequently improved nutrient digestibility when compared with pigs fed a control or antibiotic. Likewise, Ahmed et al. (2014) found that feeding dietary acidifiers in the nursery could reduce the counts of pathogenic *E. coli*, meanwhile promoting the growth of beneficial *Lactobacilli* and *Bacilli* in the gut. The current experiment did observe a main effect of day (*P* = 0.002) on nursery pig microbial populations, whereas counts of enteric bacteria were reduced from days 0 to d 21. Ultimately, further investigation is needed to adequately describe how dietary acidifiers can affect the nursery pig gut microbiome.

Blood serum parameters showed pigs consuming carbadox had lower phosphorus concentrations compared with those fed the remaining treatments. Although statistically, this difference is significant, the mean phosphorus value of pigs fed the carbadox treatment was still normal, suggesting no biological significance. Pigs fed the carbadox diet also had higher concentrations of sorbitol dehydrogenase. It is known that increased levels of sorbitol dehydrogenase can be associated with hepatocellular injury (Asada and Galambos, 1963); however, given that carbadox is metabolized by the liver and these pigs were overall healthy, this parameter could be due to drug metabolism.

In summary, this study demonstrated that feeding CTC can benefit nursery pig health and growth performance. The addition of dietary acidifiers did not alter nursery pig growth performance when compared with a control. Continued investigation into optimal inclusion levels, the mode of action and economic benefits of utilizing dietary acidifiers in place of antibiotics is warranted.
